# Timing and Type of Venous Thromboembolic Chemoprophylaxis Is Associated with Acute Traumatic Brain Injury Outcomes

**DOI:** 10.1089/neur.2022.0048

**Published:** 2022-11-10

**Authors:** Darwin Ang, Kevin Pierre, John Armstrong, James Dunne, Stephen Flaherty, Ernest Gonzalez, Mark McKenney, Patrick Offner, David Plurad, Huazhi Liu, Michele Ziglar

**Affiliations:** ^1^ Department of Trauma, HCA Florida Ocala Hospital, Ocala, Florida, USA.; ^2^ Department of Surgery, University of South Florida, Tampa, Florida, USA.; ^3^ College of Medicine, University of Central Florida, Orlando, Florida, USA.; ^4^ Department of Surgery, Medical Center, Memorial Health University, Savannah, Georgia, USA.; ^5^ Department of Trauma, Del Sol Medical Center, El Paso, Texas, USA.; ^6^ Department of Trauma, South Austin Medical Center, Austin, Texas, USA.; ^7^ Department of Trauma, Kendall Regional Medical Center, Miami, Florida, USA.; ^8^ Department of Trauma, Sky Ridge Medical Center, Lone Tree, Colorado, USA.; ^9^ Department of Trauma, Riverside Community Hospital, Riverside, California, USA.; ^10^ Clinical Services Group, Hospital Corporation of America, Nashville, Tennessee, USA.

**Keywords:** chemoprophylaxis, low-molecular-weight heparin, mortality, traumatic brain injury, unfractionated heparin, venous thromboembolic events

## Abstract

Venous thromboembolic (VTE) prophylaxis in acute traumatic brain injury (TBI) is a controversial topic with wide practice variations. This study examined the association of VTE chemoprophylaxis with inpatient mortality and VTE events among isolated TBI patients. This was a retrospective cohort study of 87 trauma centers within a large hospital system in the United States analyzing 23,548 patients with isolated TBI, 7977 of whom had moderate-to-severe TBI. Primary outcomes were inpatient mortality and VTE events. The control group received no chemoprophylaxis. Other groups received low-molecular-weight heparin (LMWH), unfractionated heparin (UFH), and combined LMWH and UFH chemoprophylaxis. Multi-variable regression accounted for confounders. Outcomes were stratified by timing of administration, body mass index (BMI), and TBI type. Patients without VTE prophylaxis had the least VTE events. LMWH had the lowest mortality for both all-isolated and moderate-to-severe isolated TBI populations at adjusted odds ratio (aOR) 0.24 (95% confidence interval [CI], 0.14–0.43) and aOR 0.25 (95% CI, 0.14–0.44), respectively. Clinically significant progression of TBI was lowest among the LMWH group (0.1%; *p* value, 0.001). After stratifying by timing of VTE chemoprophylaxis, only patients with subdural hematoma and LMWH between 6 and 24 h (*N* = 62), as well as patients with ≥35 BMI and LMWH between 6 and 24 h (*N* = 65) or >24–48 h (*N* = 54), had no VTE events. VTE chemoprophylaxis timing may have prevented VTE in certain subgroups of isolated TBI patients. Though VTE chemoprophylaxis did not prevent VTE for most TBI patients, LMWH VTE chemoprophylaxis was associated with reduced mortality.

## Introduction

Untreated venous thromboembolism (VTE) may lead to often fatal pulmonary embolisms (PEs). VTE rates are significantly higher in patients with traumatic brain injury (TBI) compared to all hospitalized patients.^1,2^ Research suggests that TBI is an independent risk factor for VTE, and VTE chemoprophylaxis may decrease overall VTE events.^3^ However, VTE prophylaxis in patients with acute TBI continues to be controversial, with wide variation in practice attributable to the concern of contributing to increasing cerebral hemorrhagic complications.^4–6^ There is emerging evidence supporting that early initiation of VTE prophylaxis in patients with TBI improves outcomes and does not lead to progressive cerebral hemorrhage.^7–9^ Current national guidelines do not provide definitive recommendations in this subgroup of trauma patients because of limited evidence regarding the specific type and timing of VTE prophylaxis.^10,11^ The goal of this study was to examine the association of type and timing of VTE chemoprophylaxis with inpatient mortality and VTE events in isolated TBI patients.

## Methods

This was a multi-center retrospective cohort study of level I, II, III, and IV community-based and academic trauma centers within a large hospital system in the United States. A total of 87 trauma centers were included from the years 2013–2018. Deep venous thrombosis (DVT) screening, TBI management, and institution of chemoprophylaxis practices varied among institutions, as determined by individual practice or local guidelines. The deidentified data set was obtained from the hospital system's administrative data set. The study was approved by the hospital system's institutional review board.

### Population

**FIG. 1.**
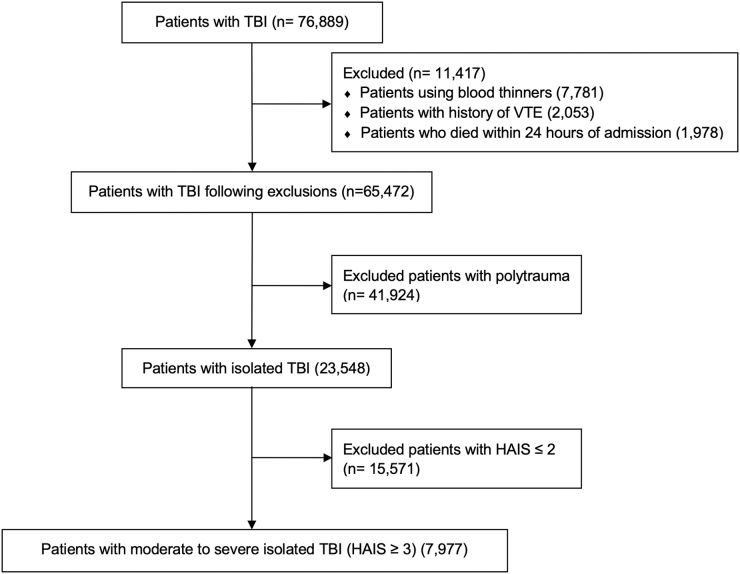
CONSORT diagram. HAIS, Head Abbreviated Injury Scores; TBI, traumatic brain injury; VTE, venous thromboembolic.

There were 76,889 patients with TBI. To avoid confounding, 11,417 patients who were treated with anticoagulants other than LMWH or UFH on an outpatient basis before admission, had a past medical history of VTE events, or died within the first 24 h of hospital admission were excluded. After these exclusions, 65,472 patients remained. Among these patients, 23,548 patients had isolated TBIs; of these, 7977 had isolated moderate-to-severe TBI with Head Abbreviated Injury Scores (HAIS) ≥3 (Fig. 1). Patients with isolated TBIs were defined as those not sustaining significant injury in other regions. TBI was defined by International Classification of Diseases, Ninth Revision (ICD-9) and International Classification of Diseases, Tenth Revision (ICD-10) codes (850-854, 959.01, S06.0-S06.9, S09.8, and S09.0). Trauma patients were identified by ICD-9 codes 800-959.9 and by ICD-10 codes S00-99, T07, T14-28, T30-34, and T79.A1-A9.

### Outcomes

Primary outcomes across groups were inpatient mortality and VTE events. VTE events were defined as either PE (415.1x, I26x, I26.90, I26.92, and I26.99) or DVT (453.4x, 453.8, I82.4x, and I82.5x) diagnosed during hospital admission and not part of the patient's past medical history. Secondary outcomes included complication rates (pneumonia, urinary tract infection [UTI], arrhythmias, sepsis, reintubation, wound infection, cardiac arrest, coagulopathy, acute respiratory distress syndrome, hospital length of stay [hLOS], intensive care unit [ICU], length of stay [LOS], and ventilator days).

### Control and exposure groups

Four groups were compared. The control group had patients who did not receive LMWH or UFH VTE chemoprophylaxis. The three groups of patients with chemoprophylaxis received LMWH only, UFH only, or both LMWH and UFH during admission. To ensure that we did not have a crossover of VTE events from the non-chemoprophylaxis group to any of the chemoprophylaxis groups, we screened for patients who were not on VTE chemoprophylaxis for 48 h after admission and who then had a VTE event with subsequent initiation of either therapeutic LMWH (2 mg/kg) or UFH (50–100 U/mL). These dosages and formulations were reviewed with our pharmacy department.

### Stratified analyses

Patients were stratified by timing of UFH or LMWH administration (before admission, <6, 6–24, >24–48, >48–72, and >72 h). The analysis was then further substratified by type of TBI and body mass index (BMI). TBIs included isolated subdural hematomas (SDHs), isolated subarachnoid hemorrhage (SAH), isolated epidural hematoma (EDH), isolated intraparenchymal hematoma (IPH), combined injuries (any combination of SDH, SAH, EDH, or IPH), and all other TBIs. BMI was stratified by <25, 25 to ≤30, 30 to ≤35, and >35.

### Statistical analysis

All data were analyzed using SAS software (version 9.4; SAS Institute Inc., Cary, NC). Parametric data expressed as proportions were evaluated by chi-square tests and the *t*-test for continuous data. Non-parametric data were evaluated by Fisher's exact test for proportions and Wilcoxon's rank-sum test for continuous data. For missing data, Rubin's multiple imputation method was performed to complete the data set.^12^ Logistic regression was used to calculate the crude univariate and adjusted multi-variate odds ratio of each exposure group when the outcome was binary. Multi-variable logistic regression was used for binary outcome variables such as mortality or the presence of VTE events. Confounders were considered in the multi-variable analysis if it was reasonable to assume, or there were published data to show, that these variables had an independent effect on mortality and VTE events in trauma patients. Hierarchical logistic regression was used for reliability adjustment to account for hospital-level variations in practice.^13,14^ The final multi-variable regression model for all groups of isolated TBI patients included age, sex, race/ethnicity, insurance status, comorbidities (Charlson comorbidity index), weight, admission Glasgow Coma Scale (GCS), HAIS score, and ICD-9 Injury Severity Score (ICISS). The final multi-variable regression model for patients with HAIS ≥3 included the same variables except for the HAIS score.

## Results

**FIG. 2.**
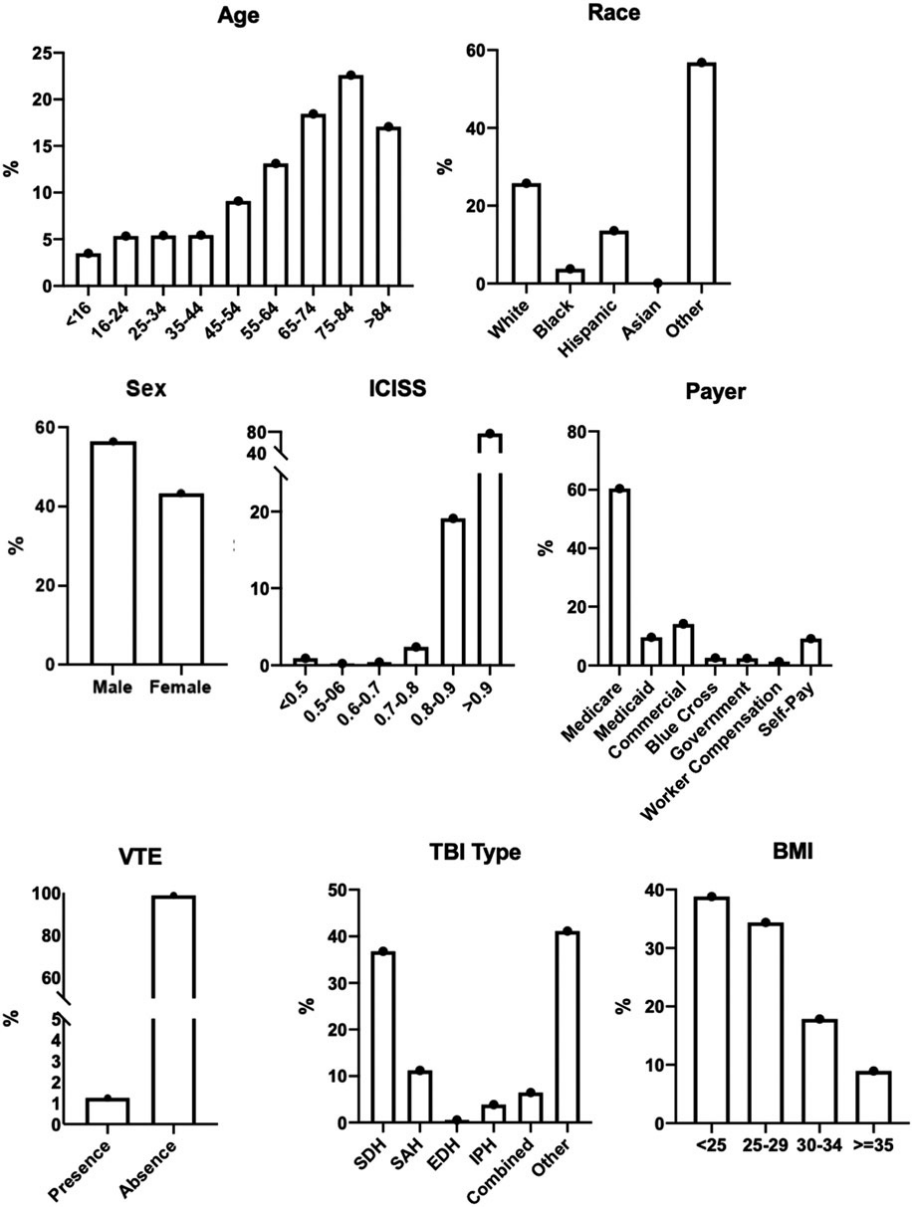
Sociodemographic distribution of isolated TBI patients. BMI, body mass index; EDH, epidural hematoma; ICISS, ICD-9 Injury Severity Score; IPH, intraparenchymal hematoma; SAH, subarachnoid hemorrhage; SDH, subdural hematoma; TBI, traumatic brain injury; VTE, venous thromboembolic.

Of 23,548 isolated TBI patients, 77% did not receive any VTE chemoprophylaxis, whereas 5430 patients received VTE chemoprophylaxis. Slightly more patients received LMWH only compared to UFH only: 2808 (11.9%) of the total isolated TBI population versus 2184 (9.3%), respectively. Our sample included a broad sociodemographic patient distribution (Fig. 2). Sociodemographic characteristics among the four groups were significantly different (see Supplementary Materials).

Patients who did not receive VTE chemoprophylaxis had the lowest number of VTE events (0.5%), whereas those who received both LMWH and UFH had the highest (12.1%), followed by UFH only (3.9%) and LMWH only (2.6%). Even after multi-variable risk adjustment, any form of VTE chemoprophylaxis had significantly higher VTE events (Table 1). Interruption of VTE chemoprophylaxis was common. For the LMWH group, 54% had VTE chemoprophylaxis stopped for ≥48 h after the initial dose. Similarly, VTE chemoprophylaxis was interrupted after the first dose for 47% of the UFH group and 77% of the combined LMWH/UFH group. The primary outcome analysis was repeated after excluding patients who received interrupted VTE chemoprophylaxis. VTE events were slightly less for the LMWH group (1.8% vs. 2.6%). However, likelihood was still higher for developing VTE (adjusted odds ratio [aOR], 5.86; 95% confidence interval [CI], 2.25–15.30) for the LMWH group. A similar result was noted for both UFH and combined groups (Table 1).

**Table 1.** Primary Outcomes for Isolated TBI and Moderate-to-Severe TBI 2013–2018

Isolated TBI	Without LMWH or UFH (*N* = 18,208)	LMWH (*N* = 2808)	UFH (*N* = 2184)	Combination LMWH/UFH (*N* = 348)
VTE (PE/DVT)	0.5%	2.6%	3.9%	12.1%
Odds ratio		5.20 (3.82, 7.08)	7.89 (5.86, 10.62)	26.74 (18.26, 39.15)
Adjusted odds ratio^*^		7.22 (3.70, 14.08)	10.63 (5.65, 20.01)	26.85 (11.80, 61.07)
aOR with reliability adjustment		7.54 (3.83, 14.86)	12.54 (6.44, 24.41)	29.91 (12.88, 69.45)
Mortality	4.2%	2.4%	8.8%	7.8%
Odds ratio		0.55 (0.42, 0.71)	2.18 (1.85, 2.57)	1.91 (1.28, 2.85)
Adjusted odds ratio^*^		0.24 (0.14, 0.42)	1.09 (0.77, 1.54)	0.42 (0.18, 0.99)
Reliability adjustment		0.24 (0.14, 0.43)	1.10 (0.77, 1.56)	0.42 (0.18, 0.99)

HAIS ≥3 (moderate-to-severe TBI)	Without LMWH or UFH (*N* = 6382)	LMWH (*N* = 700)	UFH (*N* = 767)	Combination LMWH/UFH (*N* = 128)
VTE (PE/DVT)	0.3%	2.9%	4.2%	9.4%
Odds ratio		8.91 (4.81, 16.52)	13.19 (7.57, 23.00)	31.34 (15.06, 65.19)
Adjusted odds ratio^*^		8.36 (4.01, 17.44)	11.03 (5.54, 21.98)	19.86 (7.83, 50.38)
Reliability adjustment		8.35 (4.00, 17.43)	11.22 (5.52, 22.81)	19.54 (7.60, 50.24)
Mortality	6.2%	4.1%	10.6%	8.6%
Odds ratio		0.66 (0.45, 0.97)	1.80 (1.40, 2.32)	1.43 (0.77, 2.68)
Adjusted odds ratio^*^		0.25 (0.14, 0.44)	0.99 (0.69, 1.42)	0.41 (0.17, 1.00)
aOR with reliability adjustment		0.25 (0.14, 0.44)	1.00 (0.69, 1.45)	0.41 (0.17, 1.00)

^*^ Adjusted by age, sex, race/ethnicity, weight, insurance status, ICISS, comorbidity index, GCS, and HAIS.

TBI, traumatic brain injury; VTE, venous thromboembolism; PE, pulmonary embolism; DVT, deep venous thrombosis; aOR, adjusted OR; HAIS, Head Abbreviated Injury Scores; LMWH, low-molecular-weight heparin; UFH, unfractionated heparin; ICISS, ICD-9 Injury Severity Score; GCS, Glasgow Coma Scale.

When only moderate-to-severe TBI patients (HAIS ≥3) were examined, the overall results were similar (Table 1). Patients who did not receive VTE chemoprophylaxis had the lowest rate of VTE events (0.3%), whereas for patients who received LMWH the rate was 2.9%. All groups who received VTE chemoprophylaxis had a significantly higher likelihood of VTE events, even after adjusting for confounding variables. These outcomes were still present, even when patients who had their VTE chemoprophylaxis interrupted were removed from the analysis.

Inpatient mortality rate was lowest for the LMWH group (2.4%; Table 1). Patients without VTE chemoprophylaxis had the next lowest rate (4.2%). Patients who received UFH had the highest mortality rate (8.8%). Compared to patients who received no VTE chemoprophylaxis, only the LMWH group had a significantly lower likelihood of inpatient mortality (aOR, 0.24; 95% CI, 0.14–0.43). This was true even after adjusting for injury severity, comorbidities, and admission GCS. Admission GCS was highest among patients who did not receive VTE chemoprophylaxis (13.9 ± 2.7; see Supplementary Materials). Removing patients with interrupted VTE chemoprophylaxis did not change mortality risk in any group (see Supplementary Materials).

When moderate-to-severe TBI patients (HAIS ≥3) were examined, LMWH patients continued to have the lowest mortality rate. As with the primary analysis of isolated TBI patients, those with moderate-to-severe TBI who received LMWH had a significantly lower mortality rate (aOR, 0.25; 95% CI, 0.14–0.44; Table 1). Mortality remained unchanged when patients with interrupted VTE chemoprophylaxis were removed (see Supplementary Materials).

**FIG. 3.**
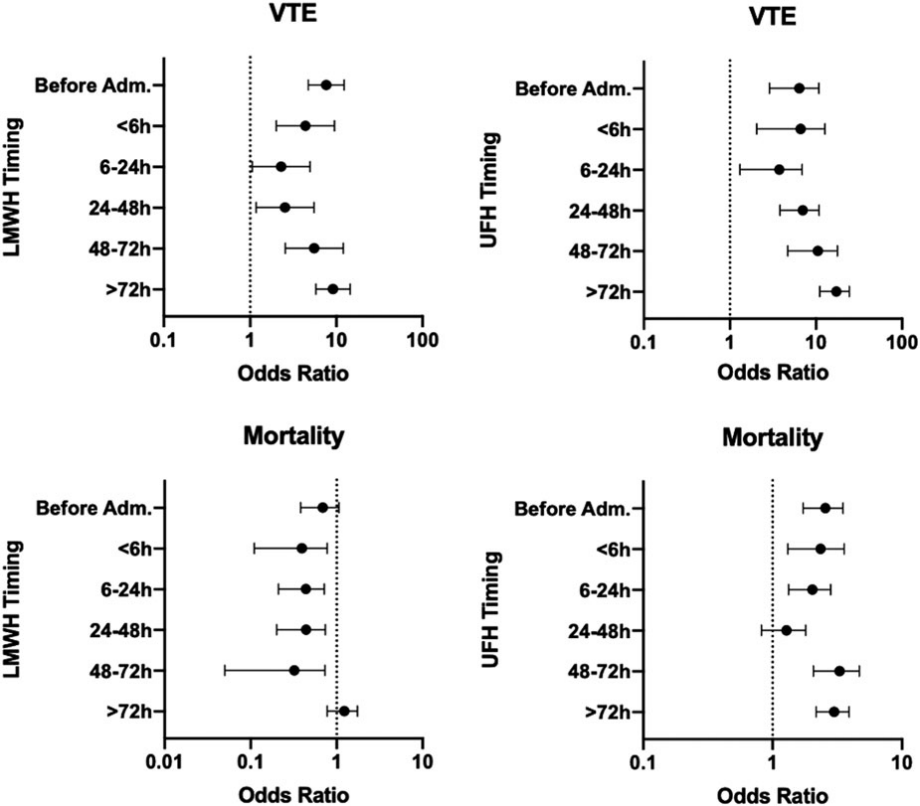
VTE outcome by timing of administration of chemoprophylaxis relative to admission for isolated TBI patients 2013–2018. TBI, traumatic brain injury; UFH, unfractionated heparin; VTE, venous thromboembolic.

Timing of VTE chemoprophylaxis was examined for its association with both VTE events and mortality. Timing of DVT prophylaxis ≥48 h after admission was associated with the highest rate of DVTs. Overall, timing of administration of VTE chemoprophylaxis did not prevent the development of VTE compared to patients without VTE chemoprophylaxis (Fig. 3). A substratified analysis was performed to examine the effect of TBI type or BMI on outcomes related to VTE chemoprophylaxis timing. For strata with ≥50 patients, only those with SDH who were given LMWH between 6 and 24 h had no VTE events (*N* = 59). When moderate-to-severe (HAIS ≥3) TBI patients were stratified by time, there were no VTE events for patients given LMWH 6–24 h after admission (*N* = 62). Patients with a BMI ≥35 had no VTE events when given LMWH 6–24 h (*N* = 65) or >24–48 h (*N* = 54). For patients who received LMWH, mortality was significantly lower than those who received no chemoprophylaxis as early as <6 h, and the benefit was still observed at 72 h (Fig. 3).

**FIG. 4.**
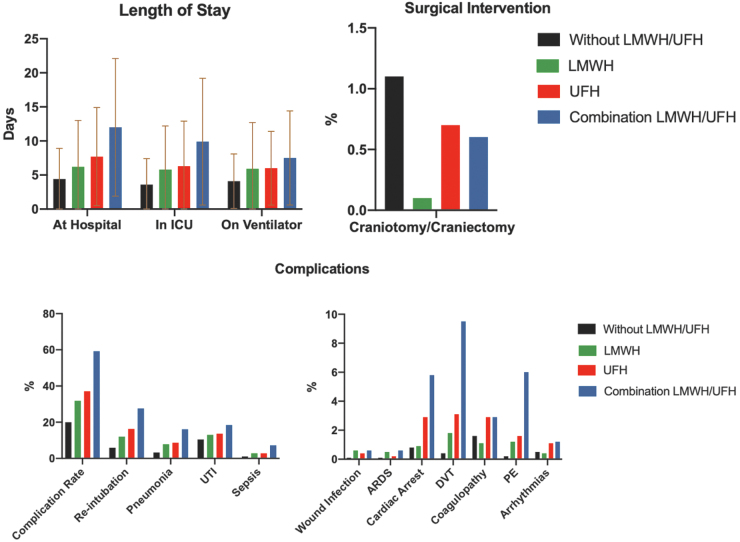
Secondary outcomes for isolated TBI patients. ARDS, acute respiratory distress syndrome; DVT, deep venous thrombosis; ICU, intensive care unit; LMHW, low-molecular-weight heparin; PE, pulmonary embolism; TBI, traumatic brain injury; UFH, unfractionated heparin; UTI, urinary tract infection.

Overall complication rates were lowest for patients who had no VTE chemoprophylaxis (20%). The most common complication among all groups was reintubation, followed by UTI and pneumonia (Fig. 4). Patients who did not receive VTE chemoprophylaxis had the lowest hLOS, ICU LOS, and ventilator days (Fig. 4). Chi-square testing revealed statistically significant differences in all groups (*p* < 0.005; see Supplementary Materials).

Patients on LMWH had the lowest frequency of craniotomy or craniectomy (0.1%). Patients without VTE chemoprophylaxis had the highest rate of craniotomy or craniectomy (1.1%). Both the UFH and combination UFH/LMWH groups were lower (0.7% and 0.6%, respectively; Fig. 4). Chi-square testing also revealed statistically significant differences (*p* < 0.005; see Supplementary Materials).

## Discussion

Previous studies have reported that TBI is an independent risk factor for VTE events.^15–18^ Yet, despite this known risk, current guidelines do not agree on the type and timing of chemoprophylaxis to prevent VTE in isolated TBI patients.^10,11^ Controversy exists because VTE chemoprophylaxis carries the potential risk of progression of intracranial hemorrhage.^6,19^ Although some studies have supported the use of either UFH or LMWH for VTE chemoprophylaxis in TBI patients,^3^ more evidence is mounting that LMWH may perform better in preventing VTE^20,21^ and convey a survival advantage over UFH among trauma^22^ and TBI^21^ patients.

All isolated TBI patients were chosen as our index population, rather than moderate-to-severe isolated TBI (HAIS ≥3) patients alone, because our goal was to examine the most likely scenario in day-to-day practice. Neither the American College of Surgeons (ACS) Trauma Quality Improvement Program (TQIP) TBI guidelines nor the Brain Trauma Foundation guidelines specify that only moderate-to-severe TBI patients should receive VTE chemoprophylaxis.^10,11^ Although many studies use HAIS as a means of identifying moderate-to-severe TBI,^3,20,23^ the HAIS is of limited clinical value for daily patient care.

Our results suggest that there may be a survival benefit among isolated TBI patients who receive LMWH as chemoprophylaxis for VTE not only relative to UFH, but also to the absence of chemoprophylaxis (aOR, 0.24; 95% CI, 0.14–0.43). The UFH group had no survival benefit over those who did not receive chemoprophylaxis. In this study, the LMWH group had the fewest craniotomies, even after receiving LMWH, compared to patients without chemoprophylaxis and the UFH group. VTE events among isolated TBI patients without chemoprophylaxis were low (0.5%). Previous studies have quoted isolated TBI VTE frequencies between 3%^3^ and 30%.^11^ Yet, the overall VTE rate for the isolated TBI population of 23,548 was also low (1.24%). The rate of VTE in the prophylactic groups was more consistent with other studies, between 2.6% and 12%. As a result, an unexpected finding of higher VTE events was found for the three groups with VTE chemoprophylaxis, even after the removal of patients who had VTE chemoprophylaxis interrupted (see Supplementary Materials). These findings were the same for moderate-to-severe isolated TBI patients as well.

In the literature, we identified one study where the DVT risk for TBI patients without VTE chemoprophylaxis was 0.4%. Similar to our results, the researchers reported higher VTE events with chemoprophylaxis and with later administration (from 3.6% to 15.4%). They concluded that, despite VTE chemoprophylaxis, TBI patients had a higher risk of forming DVT compared to trauma patients without TBI.^2^ We do not have an explanation for the low rate of VTE in the control group; however, it is likely attributable to the heterogeneous VTE risk related to the type of TBI, specific patient characteristics, and timing of administration of chemoprophylaxis.

The ACS-TQIP TBI guidelines recommend initiating VTE chemoprophylaxis within 72 h of hospital admission.^11^ Despite this, some studies have reported a low rate (e.g., 5%) of starting VTE chemoprophylaxis within day 1 of admission, improving to 30% by day 3.^24^ There are other data to suggest that the longer TBI patients are without VTE chemoprophylaxis, the higher their risk for VTE.^25^ In our initial analysis, time was not associated with a lower rate of VTE compared to patients who did not receive chemoprophylaxis. However, when we stratified by type of isolated TBI, we found that patients with SDH who were given LMWH between 6 and 24 h had no VTE events (59 patients). When moderate-to-severe (HAIS ≥3) TBI patients were stratified by time, there were no VTE events when patients were given LMWH 6–24 h after admission (*N* = 62).

When we stratified by BMI, we found that ≥35 BMI was an independent factor for improved VTE outcomes with LMWH. There were no VTE events regardless of initiation timing of LMWH after hospital admission at 6–24 h (*N* = 65) or >24–48 hours (*N* = 54). There are data supporting the use of LMWH in the morbidly obese patient^26^; however, to date, this is the first study to report a benefit of LMWH chemoprophylaxis in morbidly obese patients with TBI.

There are several limitations to this study. This is a large retrospective cohort study and not a randomized controlled trial. However, VTE events are rare, and severe TBI does not represent a large number of patients. Combining the probability of two uncommon events would require a large sample size and limits the feasibility of a prospective randomized controlled trial. Instead, we offer a population-based study specific to isolated TBI patients with no history of VTE or outpatient anticoagulation, and who survived ≥24 h. Second, we are not able to comment on progression of intracranial hemorrhage after VTE chemoprophylaxis. Instead, we have looked at indirect measurements of intracranial stability, such as the frequency of craniotomies/craniectomies after initiation of VTE chemoprophylaxis. Similar to findings in other studies, using VTE chemoprophylaxis in isolated TBI patients appears to be safe.^27^

The third limitation is that we were not able to obtain an accurate measurement of the use of mechanical compression devices (MCDs). Though their contribution to VTE chemoprophylaxis is limited, there is evidence that MCDs may contribute to VTE prevention^28^ and might be a potential confounder in our analysis. Fourth, considering that the rate of symptomatic DVT is lower than that detected by routine ultrasonography, patients with asymptomatic DVTs may have been excluded because of the lack of a routine DVT screening the involved institutions. Further, our results suggest that patients with TBI receiving VTE prophylaxis are more likely to experience VTE events compared to those with TBI not receiving VTE prophylaxis, which is contrary to most current literature that suggests that VTE prophylaxis is effective in reducing the risk of VTE.^29^ The lower rate of VTEs in patients without chemoprophylaxis in our study may simply reflect the shorter length of stay in this population.

Our study is likely not entirely reflective of real-world populations, considering that a significant number of patients were already on DVT prophylaxis before admission. Selection bias among clinicians may have resulted in a small percentage of patients receiving VTE and contributed to some of the demonstrated associations given that patients with a higher risk of death from severe TBI may have had the greatest pressure to withhold chemoprophylaxis. Similarly, the associations between mortality reduction and LMWH may have been, in part, attributable to the reluctance to initiate DVT prophylaxis in patients with TBIs known to be devastating. Last, it remains unknown whether decisions to administer VTE prophylaxis to the small number of recipients in this study received were based on reasons unrelated to TBI.

Despite these limitations, this study does represent one of the largest populations of isolated TBIs with respect to VTE chemoprophylaxis and its timing. The large population allowed for the study of smaller increments of time, TBI types, and comorbidities. Importantly, the use of the clinical data warehouse facilitated the assessment of TBI-specific factors related to severity of injury and admission GCS at a population level.

The results of this population-based study raise two questions. First, is an isolated TBI truly an independent risk factor for VTE? Acute TBI pathophysiology includes both hyper- and hypocoagulable states. The mechanism of coagulopathy after TBI is poorly defined, and the clinical significance of coagulopathy after TBI is not clearly elucidated.^30^ Clinically, though we did find a benefit for LMWH VTE chemoprophylaxis among certain subpopulations of TBI patients (i.e., SDH and ≥35 BMI), this was not true for most isolated TBI patients. Other studies have also not shown an increased risk of VTE among patients with TBI, but these were polytrauma patients.^31^ Second, should the purpose of VTE chemoprophylaxis be reconsidered? LMWH chemoprophylaxis compared with the absence of VTE chemoprophylaxis was associated with decreased mortality in all isolated and moderate-to-severe TBI, and lower mortality rates were observed with its administration at ≤72 h. Early evidence from basic science and clinical studies suggests that LMWH may have neuroprotective effects.^32–40^ If LMWH does not prevent VTE, perhaps it has a role in reducing mortality in isolated TBI patients. Future similar studies that also examine cause of mortality may help answer these questions.

## Conclusion

For isolated TBI patients, regardless of severity, LMWH VTE chemoprophylaxis was associated with reduced mortality. Though timing of VTE chemoprophylaxis may have prevented VTE in certain subgroups of patients, this study did not find that VTE chemoprophylaxis prevented VTE for the majority of isolated TBI patients.

Use of LMWH was not associated with the clinically significant progression of TBI that led to operative interventions. Coupled with lower associated mortality; the use of LMWH appears to be preferable to UFH and no VTE chemoprophylaxis for isolated TBI patients.

## Supplementary Material

Supplemental data

## Abbreviations Used

ACS: American College of Surgeons
aOR: adjusted odds ratio
BMI: body mass index
CI: confidence interval
DVT: deep venous thrombosis
EDH: epidural hematoma
GCS: Glasgow Coma Scale
HAIS: Head Abbreviated Injury Scores
hLOS: hospital length of stay
ICD-9: International Classification of Diseases, Ninth Revision
ICD-10: International Classification of Diseases, Tenth Revision
ICISS: ICD-9 Injury Severity Score
ICU: intensive care unit
IPH: intraparenchymal hematoma
LMWH: low-molecular-weight heparin
LOS: length of stay
MCDs: mechanical compression devices
PE: pulmonary embolus
SAH: subarachnoid hemorrhage
SDHs: subdural hematomas
TBI: traumatic brain injury
TQIP: Trauma Quality Improvement Program
UFH: unfractionated heparin
UTI: urinary tract infection
VTE: venous thromboembolic
