# Supplementary material for: Timing and Type of Venous Thromboembolic Chemoprophylaxis Is Associated with Acute Traumatic Brain Injury Outcomes
Source: Neurotrauma Rep. 2022 Nov 10;3(1):511–21. doi: 10.1089/neur.2022.0048 (PMC9718434; doi:10.1089/neur.2022.0048)
Supplement: Supplemental data [file Suppl_Data.docx]

**Supplementary table 1. Sociodemographic and Secondary Outcomes for Isolated TBI 2013-2018**

|  | **Without**  **LMWH/UFH**  **(N=18,208)** | **LMWH (N=2,808)** | **UFH (N=2,184)** | **Combination LMWH/UFH (N=348)** | **p-value** |
| --- | --- | --- | --- | --- | --- |
| **Age** |  |  |  |  |  |
| <16 | 4.0% | 0.6% | 3.4% | 0.9% | <0.0001 |
| 16-24 | 5.1% | 9.3% | 2.7% | 2.6% | 0.15 |
| 25-34 | 5.1% | 9.0% | 3.3% | 5.5% | 0.68 |
| 35-44 | 5.1% | 8.4% | 4.6% | 5.5% | 0.05 |
| 45-54 | 8.5% | 12.7% | 8.9% | 13.5% | <0.0001 |
| 55-64 | 12.6% | 15.0% | 14.4% | 17.0% | <0.0001 |
| 65-74 | 18.2% | 17.5% | 21.3% | 21.6% | 0.002 |
| 75-84 | 23.2% | 16.1% | 26.5% | 21.0% | 0.51 |
| >84 | 18.3% | 11.5% | 14.9% | 12.6% | <0.0001 |
| **Gender** |  |  |  |  |  |
| Male | 56.0% | 58.7% | 58.0% | 61.7% | 0.001 |
| Female | 44.0% | 41.3% | 42.0% | 38.3% | 0.001 |
|  |  |  |  |  |  |
| **Race** |  |  |  |  |  |
| White | 25.1% | 24.2% | 32.7% | 28.5% | <0.0001 |
| Black | 3.6% | 4.5% | 4.2% | 2.9% | 0.13 |
| Hispanic | 13.7% | 13.9% | 12.3% | 11.8% | 0.10 |
| Asian | 0.1% | 0.2% | 0.1% | 0.0% | 0.76 |
| Other | 57.5% | 57.2% | 50.8% | 56.9% | <0.0001 |
| **Payer** |  |  |  |  |  |
| Medicare | 61.1% | 50.8% | 67.2% | 60.3% | 0.63 |
| Medicaid | 9.6% | 11.1% | 8.3% | 10.6% | 0.75 |
| Commercial | 13.7% | 18.3% | 13.6% | 12.9% | 0.05 |
| Blue Cross | 2.7% | 2.9% | 1.2% | 4.3% | 0.03 |
| Government | 2.6% | 1.9% | 2.5% | 1.7% | 0.12 |
| Worker Compensation | 1.3% | 2.4% | 0.7% | 2.0% | 0.52 |
| Self-Pay | 9.0% | 12.8% | 6.5% | 8.1% | 0.28 |
| **ICISS** |  |  |  |  |  |
| <0.5 | 0.9% | 0.4% | 1.4% | 2.9% | 0.02 |
| 0.5-06 | 0.2% | 0.3% | 0.5% | 0.3% | 0.003 |
| 0.6-0.7 | 0.3% | 0.6% | 1.1% | 1.7% | <0.0001 |
| 0.7-0.8 | 2.1% | 3.0% | 3.9% | 4.3% | <0.0001 |
| 0.8-0.9 | 19.0% | 16.6% | 22.1% | 27.0% | 0.001 |
| >0.9 | 77.5% | 79.1% | 71.1% | 63.8% | <0.0001 |
| **VTE** |  |  |  |  |  |
| presence | 0.5% | 2.6% | 3.9% | 12.1% | <0.0001 |
| absence | 99.5% | 97.4% | 96.1% | 87.9% | <0.0001 |
|  |  |  |  |  |  |
| **TBI Type** |  |  |  |  |  |
| Subdural | 39.2% | 21.4% | 37.7% | 30.2% | <0.0001 |
| Subarachnoid | 12.0% | 6.0% | 11.8% | 8.6% | <0.0001 |
| Epidural | 0.6% | 0.3% | 0.5% | 0.3% | 0.07 |
| Intraparenchymal Hemorrhage | 4.3% | 1.5% | 3.1% | 4.0% | <0.0001 |
| Combined | 6.5% | 5.1% | 7.3% | 6.9% | 0.81 |
| All Others | 37.3% | 65.8% | 39.6% | 50.0% | <0.0001 |
| **BMI** |  |  |  |  |  |
| <25 | 39.3% | 37.9% | 36.9% | 33.1% | 0.001 |
| 25-29 | 34.1% | 35.7% | 34.3% | 39.4% | 0.11 |
| 30-34 | 17.7% | 17.3% | 19.8% | 19.0% | 0.05 |
| >=35 | 8.9% | 9.1% | 9.1% | 8.6% | 0.87 |
|  |  |  |  |  |  |
| **Admission GCS** | 13.9(±2.7) | 13.0 (±3.6) | 12.7 (±4.0) | 11.9 (±4.4) | <0.0001 |
| **Craniotomy/Craniectomy** | 1.1% | 0.1% | 0.7% | 0.6% | 0.001 |
| **Hospital LOS** | 4.4 (±4.5) | 6.2 (±6.8) | 7.7 (±7.2) | 12.0 (±10.1) | <0.0001 |
| **ICU LOS** | 3.6(±3.8) | 5.8 (±6.4) | 6.3 (±6.6) | 9.9 (±9.3) | <0.0001 |
| **Vent Days** | 4.1 (±4.0) | 5.9 (±6.8) | 6.0 (±5.4) | 7.5 (±6.9) | <0.0001 |
| **VTE prophylaxis interrupted for >48 hours after first use** |  | 54.4% | 46.7% | 77.0% | <0.0001 |
| **Complications Rate** | 20.0% | 31.8% | 37.1% | 59.2% | <0.0001 |
| ARDS | 0.1% | 0.5% | 0.2% | 0.6% | 0.004 |
| Pneumonia | 3.3% | 7.8% | 8.7% | 16.1% | <0.0001 |
| UTI | 10.5% | 13.0% | 13.7% | 18.4% | <0.0001 |
| Arrhythmias | 0.5% | 0.4% | 1.1% | 1.2% | 0.004 |
| Sepsis | 1.1% | 2.8% | 2.8% | 7.2% | <0.0001 |
| Re-intubation | 5.9% | 12.0% | 16.3% | 27.6% | <0.0001 |
| Wound Infection | 0.1% | 0.6% | 0.4% | 0.6% | <0.0001 |
| Cardiac Arrest | 0.8% | 0.9% | 2.9% | 5.8% | <0.0001 |
| DVT | 0.4% | 1.8% | 3.1% | 9.5% | <0.0001 |
| Coagulopathy | 1.6% | 1.1% | 2.9% | 2.9% | 0.002 |
| PE | 0.2% | 1.2% | 1.6% | 6.0% | <0.0001 |

**Supplementary table 2. Primary Outcomes for Isolated TBI and Moderate to Severe TBI without interruption of VTE Chemoprophylaxis, 2013-2018**

| **Isolate TBI** | **Without**  **LMWH or UFH (N=18,208)** | **LMWH (N=1,281)** | **UFH (N=1,163)** | **Combination LMWH/UFH (N=80)** |
| --- | --- | --- | --- | --- |
| **VTE (PE/DVT)** | 0.5% | 1.8% | 4.1% | 13.8% |
| Odds Ratio |  | 3.56 (2.25, 5.64) | 8.39 (5.89, 11.94) | 31.05 (15.92, 60.57) |
| Adjusted Odds Ratio* |  | 5.45 (2.10, 14.13) | 14.72 (7.36, 29.45) | 17.75 (3.60, 87.52) |
| Reliability Adjustment |  | 5.86 (2.25, 15.30) | 18.20 (8.38, 39.52) | 19.25 (3.86, 95.94) |
| **Mortality** | 4.2% | 2.7% | 9.5% | 3.8% |
| Odds Ratio |  | 0.64 (0.45, 0.90) | 2.38 (1.93, 2.93) | 0.89 (0.28, 2.81) |
| Adjusted Odds Ratio* |  | 0.31 (0.13, 0.74) | 1.10 (0.68, 1.76) | 0.16 (0.02, 1.39) |
| Reliability Adjustment |  | 0.31 (0.13, 0.74) | 1.10 (0.69, 1.77) | 0.16 (0.02, 1.41) |
|  |  |  |  |  |
| **HAIS ≥3 (Moderate to Severe TBI)** | **Without**  **LMWH/UFH**  **(N=6,382)** | **LMWH (N=269)** | **UFH(N=411)** | **Combination LMWH/UFH (N=25)** |
| **VTE (PE/DVT)** | 0.3% | 1.9% | 5.4% | 4.0% |
| Odds Ratio |  | 5.74 (2.15, 15.33) | 17.13 (9.34, 31.42) | 12.62 (1.63, 97.62) |
| Adjusted Odds Ratio* |  | 4.75 (1.52, 14.89) | 15.45 (7.24, 32.96) | 8.89 (1.03, 77.06) |
| Reliability Adjustment |  | 4.71 (1.49, 14.94) | 16.77 (7.17, 39.21) | 9.28 (1.09, 79.10) |
| **Mortality** | 6.2% | 5.2% | 11.7% | 8.0% |
| Odds Ratio |  | 0.84 (0.48, 1.45) | 2.02 (1.47, 2.77) | 1.33 (0.31, 5.64) |
| Adjusted Odds Ratio* |  | 0.39 (0.16, 0.93) | 1.03 (0.63, 1.67) | 0.07 (0.004, 1.38) |
| Reliability Adjustment |  | 0.39 (0.16, 0.93) | 1.03 (0.63, 1.69) | 0.07 (0.004, 1.34) |

* Adjusted by age, gender, race, weight, insurance status, ICISS, comorbidity index, GCS, and HAIS

**Supplementary table 3. VTE Outcome by Timing of Administration of Chemoprophylaxis after Admission for Isolated TBI Patients 2013-2018**

| **All Isolated TBI Patients** | **VTE** | Odds Ratio  OR (95% CI) | Adjusted Odds Ratio*  aOR (95% CI) |
| --- | --- | --- | --- |
| **Without LMWH/UFH N=18,208** | 0.5% |  |  |
| **LMWH Before Admission (N=557)** | 3.8% | 7.63 (4.72, 12.35) | 8.89 (1.97, 40.13) |
| **<6 Hrs After Admission (N=319)** | 2.2% | 4.37 (2.01, 9.50) | 5.66 (0.72, 44.81) |
| **6-24 Hrs After Adm. (N=602)** | 1.2% | 2.29 (1.06, 4.96) | 2.52 (0.33, 19.62) |
| **24-48 Hrs After Adm. (N=543)** | 1.3% | 2.54 (1.17, 5.51) | 1.68 (0.22, 12.80) |
| **48-72 Hrs After Adm. (N=253)** | 2.8% | 5.54 (2.55, 12.07) | 5.36 (1.21, 23.64) |
| **>72 Hrs After Adm. (N=534)** | 4.5% | 9.17 (5.80, 14.48) | 13.34 (6.13, 29.00) |
| **UFH Before Admission (N=360)** | 2.8% | 5.57 (2.87, 10.78) | 0.01 (<0.001, >999) |
| **<6 Hrs After Admission (N=196)** | 2.6% | 5.10 (2.05, 12.68) | 0.01 (<0.001, >999) |
| **6-24 Hrs After Adm. (N=395)** | 1.5% | 3.01 (1.31, 6.90) | 5.59 (1.26, 24.78) |
| **24-48 Hrs After Adm. (N=532)** | 3.2% | 6.43 (3.81, 10.86) | 5.72 (1.88, 17.39) |
| **48-72 Hrs After Adm. (N=224)** | 4.5% | 9.10 (4.68, 17.72) | 9.15 (2.57, 32.63( |
| **>72 Hrs After Adm. (N=476)** | 7.8% | 16.42 (11.09, 24.31) | 28.01 (13.52, 58.03) |
| **Combined LMWH/UFH Before Admission (N=81)** | 13.6% | 30.61 (15.70, 59.67) | 63.60 (11.85, 341.48) |
| **<6 Hrs After Admission (N=36)** | 16.7% | 38.96 (15.84, 95.81) | 0.01 (<0.001, >999) |
| **6-24 Hrs After Adm. (N=54)** | 5.6% | 11.46 (3.51, 37.37) | 25.12 (3.03, 208.63) |
| **24-48 Hrs After Adm. (N=54)** | 7.4% | 15.58 (5.52, 44.03) | 10.90 (1.36, 87.37) |
| **48-72 Hrs After Adm. (N=35)** | 14.3% | 32.47 (12.33, 85.51) | 48.27 (9.33, 249.66) |
| **>72 Hrs After Adm. (N=88)** | 14.8% | 33.77 (18.11, 62.96) | 30.77 (10.26, 92.30) |
|  | **Mortality** | Odds Ratio | Adjusted Odds Ratio* |
| **Without LMWH/UFH (N=18,208)** | 4.2% |  |  |
| **LMWH Before Admission (N=557)** | 2.7% | 0.63 (0.38, 1.06) | <0.001 (<0.001, >999) |
| **<6 Hrs After Admission (N=319)** | 1.3% | 0.29 (0.11, 0.78) | 0.002 (<0.001, >999) |
| **6-24 Hrs After Adm. (N=602)** | 1.7% | 0.38 (0.21, 0.72) | 0.31 (0.04, 2.42) |
| **24-48 Hrs After Adm. (N=543)** | 1.7% | 0.38 (0.20, 0.74) | 0.21 (0.05, 0.99) |
| **48-72 Hrs After Adm. (N=253)** | 0.8% | 0.18 (0.05, 0.73) | 0.07 (0.01 0.59) |
| **>72 Hrs After Adm. (N=534)** | 4.9% | 1.16 (0.78, 1.74) | 0.34 (0.17, 0.65) |
| **UFH Before Admission (N=360)** | 9.7% | 2.45 (1.72, 3.50) | 1.35 (0.48, 3.77) |
| **<6 Hrs After Admission (N=196)** | 8.7% | 2.16 (1.31, 3.57) | 0.40 (0.06, 2.78) |
| **6-24 Hrs After Adm. (N=395)** | 7.9% | 1.94 (1.33, 2.81) | 1.80 (0.85, 3.85) |
| **24-48 Hrs After Adm. (N=532)** | 5.1% | 1.22 (0.82, 1.80) | 0.65 (0.32, 1.31) |
| **48-72 Hrs After Adm. (N=224)** | 12.1% | 3.12 (2.07, 4.69) | 1.65 (0.79, 3.42) |
| **>72 Hrs After Adm. (N=476)** | 11.3% | 2.91 (2.17, 3.90) | 1.06 (0.60, 1.88) |
| **Combined LMWH/UFH Before Admission (N=81)** | 7.4% | 1.82 (0.79, 4.19) | 0.13 (0.004, 3.51) |
| **<6 Hrs After Admission (N=36)** | 13.9% | 3.67 (1.42, 9.46) | <0.001 (<0.001, >999) |
| **6-24 Hrs After Adm. (N=54)** | 7.4% | 1.82 (0.66, 5.05) | 1.27 (0.12, 13.11) |
| **24-48 Hrs After Adm. (N=54)** | 3.7% | 0.88 (0.21, 3.60) | 0.59 (0.12, 2.91) |
| **48-72 Hrs After Adm. (N=35)** | 8.6% | 2.13 (0.65, 6.98) | 0.44 (0.05, 4.30) |
| **>72 Hrs After Adm. (N=88)** | 8.0% | 1.97 (0.91, 4.27) | 0.33 (0.09, 1.20) |

* Adjusted by age, gender, race, weight, insurance status, ICISS, comorbidity index, GCS, and HAIS
